# AIDS and the pancreas in the HAART era: a cross sectional study

**DOI:** 10.1186/1755-7682-6-28

**Published:** 2013-07-15

**Authors:** Alexandre G Barbosa, Ethel Z Chehter, Marcelo R Bacci, Ana A Mader, Fernando LA Fonseca

**Affiliations:** 1 Department of Gastroenterology, Faculdade de Medicina do ABC, Santo André, Brazil; 2 Department of General Practice, Faculdade de Medicina do ABC, Santo André, Brazil; 3 Department of Pathology, Faculdade de Medicina do ABC, Santo André, Brazil

**Keywords:** HIV, Exocrine pancreatic insufficiency, Highly active anti-retroviral therapy, Chronic pancreatitis

## Abstract

**Backgrounds:**

The aim of this study is identify the main morphological patterns of the pancreas in AIDS patients in use of Higly Active Antiretorviral Therapy (HAART).

**Methods:**

We conducted a cross sectional study in the year of 2010. The inclusion criteria were patients older than 18 years who died of AIDS with the use of HAART (2006–2009) and underwent to autopsy . They were compared with a group of 109 patients who died of AIDS in 1995 before the HAART therapy. All the autopsies were made in the Death Verification Service of São Paulo.

**Results:**

The HAART group presented pancreas abnormalities lighter than no HAART users. In the HAART group, histology shows: reduction of zymogen granules in the acinar cells (ZG) higher percentage of cases, “dysplasia-like” presents lower and pancreatic acinar atrophy, presents higher percentage of cases compared to no HAART group. The exocrine pancreas in treated patients was distinguished by the high level of atrophy, sharp reduction of zymogen granules and high level of apoptosis, reflecting degeneration and lower level of protein-caloric malnutrition.

**Conclusions:**

The islets of Langerhans in HAART group were increased in number and volume and with high level of nuclear dysplasia. The antiviral therapy and a longer survival resulted in a higher atrophy and reduction of enzymes, increasing the apoptosis and generated important changes in the pancreatic islets, probably resulting in clinical laboratory repercussion.

We found no evidence of pancreatic histopathological lesions secondary to antiretroviral therapy.

## Backgrounds

AIDS is a pandemic disease that affects over 34 million people worldwide [1]. There are 1,4 million people infected with HIV in Latin America. In Brazil there are about 590,000 infected people, and the highest concentration of infected cases is in the state of São Paulo, with 344,150, followed by the southern region of the country with 115,598 [2].

Until the year 2000, pancreatic impairment in HIV patients varied from 11 to 65%. However, the majority of studies are retrospective with histological data rarely reported [3-6]. We found no systematic prospective study of pancreatic disease in AIDS reported in the literature.

The pancreatic changes in patients infected with HIV may be due to causes unrelated to AIDS, such as alcoholism, diabetes, cholelithiasis, adenocarcinoma, acute pancreatitis, chronic pancreatitis and drugs adverse effects [5-9]. Yet, causes related to AIDS include opportunistic infections and drugs used in its treatment, such as asparaginase, azathioprine among others [5-9].

Chehter *et al.* found in patients, who had died of AIDS without antiretroviral treatment, pancreatic abnormalities due to protein-caloric malnutrition. The rationale for these alterations was: malnutrition, weight loss and wasting syndrome. The wasting syndrome associated factors found in this study were: anorexia, dysphagia, odynophagia, drugs, neurological factors, diarrhea, pancreatic enzyme deficiency, increased muscle protein, hyper metabolism and increase of cytokines. The microscopic histological aspects revealed decrease in the zymogen granules in the pancreatic acinar cells, parenchymal atrophy, steatosis, double contour core, pycnotic nuclear changes and also some irregular nuclear patterns defined as “dysplasia-like” were highlighted [5,6].

These findings may suggest a clinical correlation with exocrine pancreatic insufficiency. Would it be possible?

The objective of this study is to compare the morphological patterns in pancreatic histology in patients who in 1995 died of AIDS without using antiretroviral drugs with patients who died of the same cause and used this therapy in 2010.

## Methods

A cross-sectional study was conducted with 20 patients diagnosed with AIDS from June 2006 to December 2009. They received HAART treatment and underwent necropsy by the Death Verification Service of São Paulo – University of São Paulo. They were compared with a group of 109 adult patients, who had died of AIDS in 1995 without using highly active antiretroviral treatment (HAART), in order to identify the changes that occured by the treatment used. From the 109 patients only 39 used the former treatment with AZT.

The inclusion criteria for the HAART group (2010) were: patients older than 18 years, who died of AIDS using antiretroviral drugs and underwent autopsy.

The author’s data bank was used in order to collect the data of the pre-HAART group, published in 2000 [6].

This study was made with the collaboration of the Pathology Department of the Faculdade de Medicina do ABC, and approved by the ethics committee of the same institution (registered number 141/2006).

The exclusion criteria were patients younger than 18 years of age and the absence of use of antiretroviral drugs for the HAART group.

The data of HAART group was obtained from medical records and autopsy reports. The data of non-HAART group was obtained from the author’s data bank [6].

The categoric variables chosen to compare the groups were age, sex, race, body mass index (BMI), alcoholism, smoking and HIV treatment.

Alcoholism was defined by the consumption of more than 50g of alcohol in a weekly routine. Smoking was defined by the consumption of at least 10 tobacco cigarettes in a regular basis. The regular HIV drugs used in the HAART group were protease inhibitors, non-nucleoside reverse transcriptase inhibitors and nucleoside reverse transcriptase inhibitors.

All HIV positive cases were autopsied by the Death Verification Service of São Paulo by a single experienced pathologist. In the macroscopic analysis, the pancreas was removed entirely. Its weight in grams and the presence of abnormalities (hemorrhage, necrosis, fibrosis and tumor) were registered.

The analysis proceeded to a sequential transversal sectioning of the pancreas with a sample from the head, body and tail. The fixation of the samples was made with 10% buffered formalin. They were then dehydrated in graded alcohols and cleared in xylene for the final paraffin immersion.

The paraffin blocks were submitted to histological sections of 4 μm, placed on slides and stained by standard hematoxylin-eosin technique (HE). Finally, the slides were added on glass coverlips coated with ointment.

The histopathological analysis was performed using a Nikon binocular microscope (Tokyo,Japan) with five achromatic objective lens [10,11].

The analysis included: acinar cells and lumen, interlobular and intralobular pancreatic ducts (dilation and protein plugs in the lumina), abnormalities in the stroma (presence or absence of inflammatory infiltrate, edema, hemorrhage, fibrosis and steatosis) and islets of Langerhans.

The abnormalities found were quantified by semi-quantitative method with the following rating scale: 0 = absent, 1 = mild, 2 = moderate and 3 = intense. In the acinar cell the zymogen granules in the cytoplasm was evaluated as well as the presence or absence of lesions, such as the dysplasia-like lesions of the core and apoptosis. It was considered “dysplasia-like” when the acinar cell core was enlarged, hyperchromatic with dense and irregular chromatin.

For apoptosis, cells were positively identified by the criteria of Kerr [10]. They were those with eosinophilic cytoplasm and chromatin condensation, presence of nuclear fragmentation without inflammatory reaction around. The size of the acinar cell, changes in number and volume of islets, as well as changes in the type of dysplasia-like lesions of the core were evaluated. In the stroma the degrees of pancreatic steatosis, inflammation, hemorrhage, edema, fibrosis, presence or absence of located lesions, such as necrosis, granulomas, abscesses, calcifications and neoplasias, were evaluated.

The pancreatic samples were analyzed through a semi-quantitative scale used in order to settle some parameters of the acinar cells and pancreatic stroma.

The scale used is listed below

– level 0: absence of the observed parameter in 10 “fields” × 400

– level 1: presence of the parameter observed in less than 25% of the 10 fields

– level 2: presence of the parameter observed between 25 and 50% of the 10 fields

– level 3: presence of the parameter observed in more than 50% of the 10 fields

The purpose of this scale was searching for the reduction of zymogen granules, reduction of acinar cells (atrophy), pancreatic steatosis and nucleus abnormalities (hipercromatosis, dense cromatine, double contour, schrunken).

Other pancreatic strutures were analyzed in a qualitative basis (normal or abnormal). This analysis included the acinar lumen, centroacinar cell, vessels, nerves and Langerhan’s islets [6].

Pancreatitis was defined according to the 1992 Atlanta classification [12,13].

It is characterized as an inflammatory and necrotic process of abrupt onset, whose morphological changes range from interstitial edema and minimal histological changes to large confluent areas of necrosis and hemorrhage. Initially, the center of the lesion is located around the steatonecrosis perilobular, extending to the vessels, acinar cells and ducts.

The focus of lymphocytic inflammatory infiltrate was characterized as an aggregate of lymphocytes and/or plasma cells, unifocal or multifocal in the stroma, as a consequence of the inflammatory process in the pancreas. Its progression can result in healing process and loss of exocrine and endocrine pancreatic functions.

For the evaluation of fibrosis, besides the usual HE staining, an analysis by the histochemical technique of Masson’s trichrome was also carried out [10,11]. For the evaluation of opportunistic microorganisms, such as fungi and alcohol-acid resistant bacilli, both the PAS histochemical and the Fite-Faraco techniques were respectively applied [10,11].

The immunohistochemical analysis was performed for the monoclonal mouse anti-cytomegalovirus antibody (CMV – clone MO854 – Dako) and the monoclonal mouse anti-pneumocystis carinii antibody (PMC- clone MOB 091–05 Dako) [10,11].

Student’s *t* test was used to compare the average of the two groups. In order to test the homogeneity between the ratios, either the chi-square test or Fisher’s exact test was used when there were expected frequencies less than 5. The significance level used for the tests was 5%.

## Results

In Table 1 we observe that the groups differ (p<0.001) in relation to the weight of the pancreas, and the difference between them is statistically significant: 20 cases in the 2010 group (98.75 g) and 109 cases in the 1995 group (148.80 g) (Table 1).

**Table 1:** AIDS: anthropometric data and parameter of the pancreas in 1995 (n = 109) and 2010 (n = 20)

**Variable**	**Category**	**1995 (n=109)**	**2010 (n=20)**	**p**
Age (years)		37,39* (±10,09)**	40,35* (±8,87)**	0,223^1^
Pancreas Weight (g)		138,80* (±33,10)**	98,75* (±31,92)**	< 0,001^1^
BMI		20,68* (±3,28)**	20,70* (±3,28)**	0,981^1^
Sex	Male	80 (73,4%)	17 (85%)	0,400^2^
Ethnicity	White	66 (60,6%)	13 (65%)	0,818^3^
	Black	43 (26,6%)	7 (20%)	

(1) Descriptive level of probability of the Student *t* test.

(2) Descriptive level of probability of Fisher’s exact test.

(3) Descriptive level of probability of the chi-square.

* Average.

** Standard deviation.

In Table 2 we observe that the groups differ (p<0.001) in relation to the presence of fever, abdominal pain, diabetes, alcoholism, HAART (NRTI, NNRTI and PI) AZT and smoke. Fever was more frequent in the non-HAART group (p<0.001).

**Table 2:** General clinical manifestations, alcoholism, smoking, and pancreatic manifestations of drug use in the treatment of AIDS in 1995(n= 109) and 2010 (n = 20)

**Signs and symptoms**	**1995 (n=109)**		**2010 (n=20)**		**p**
	**n**	**%**	**n**	**%**	
Fever	99	90,8	4	20,0	< 0,001^(1)^
Thinning	40	36,7	12	60,0	0,051 ^(2)^
Diarrhoea	36	33,0	8	40,0	0,545^ (2)^
Abdominal pain	11	10,1	6	30,0	0,027 ^(1)^
Alcoholics	23	21,1	18	90,0	< 0,001^(2)^
Smoke	29	26,6	15	75,0	< 0,001^(2)^
Diabetes	0	0,0	2	10,0	0,023 ^(1)^
ITRN (average of three years)	0	0,0	19	95,0	< 0,001^(1)^
AZT (average of three years)	39	35,8	18	90,0	< 0,001^(2)^
ITRnN (average of three years)	0	0,0	2	10,0	0,023 ^(1)^
IP (average of three years)	0	0,0	3	15,0	0,003 ^(1)^

(1) Descriptive level of probability of Fisher’s exact test.

(2) Descriptive level of probability of the chi-square.

**Figure 1:**
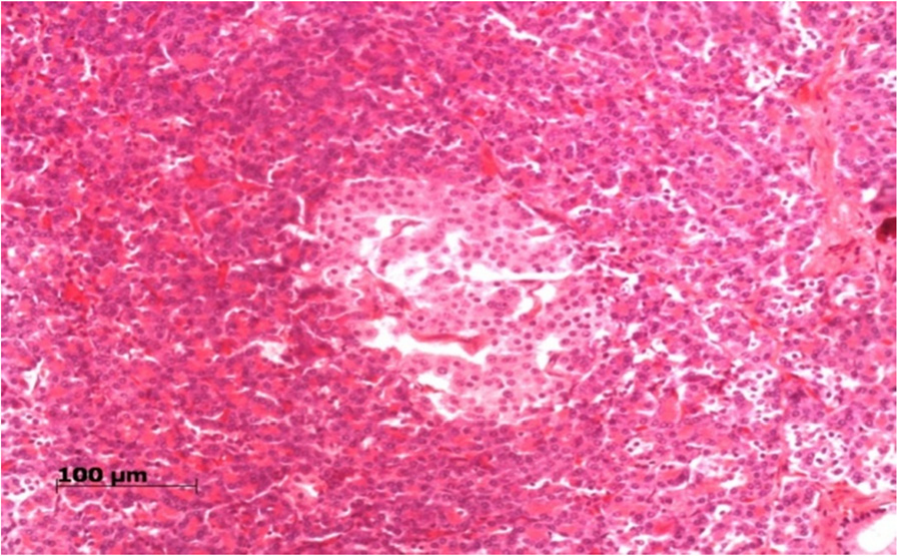
(HE 200x): Increased size of the islet of Langerhans.

In Table 3 we observe a difference in the frequency of zymogen decrease granules: the HAART group comparatively presented 1 case with no alterations, and 9, 6 and 4 mild, moderate and intense degree cases respectively (Figure 1).

**Table 3:** Microscopic features semi-quantitative components of acinar, stromal pancreatic histology of acinar, ducts and pancreatic lesions located in degrees in the year 1995 (n= 109) and 2010 (n = 20)

		**Degree**				
**Variable**	**Year**	**Absent**	**Light**	**Moderate**	**Intense**	p*
Decreased zimogen granula	1995	52 (47,7%)	52 (47,7%)	5 (4,6%)	0 (0,0%)	< 0,001
	2010	1 (5,0%)	9 (45,0%)	6 (30,0%)	4 (20,0%)	
Dysplasia-like	1995	35 (32,1%)	48 (44,0%)	19 (17,4%)	7 (6,4%)	< 0,001
	2010	19 (95,0%)	0 (0,0%)	0 (0,0%)	1 (5,0%)	
Atrophy	1995	44 (40,4%)	45 (41,3%)	17 (15,6%)	3 (2,8%)	0,017
	2010	2 (10,0%)	13 (65,0%)	3 (15,0%)	2 (10,0%)	
Steatosis	1995	36 (33,0%)	43 (39,5%)	23 (21,1%)	7 (6,4%)	0,689
	2010	7 (35,0%)	7 (35,0%)	6 (30,0%)	0 (0,0%)	
Inflammation	1995	59 (54,1%)	39 (35,8%)	7 (6,4%)	4 (3,7%)	0,673
	2010	12 (60,0%)	5 (25,0%)	2 (10,0%)	1 (5,0%)	
Haemorrhage	1995	78 (71,6%)	26 (23,9%)	4 (3,7%)	1 (0,9%)	0,921
	2010	14 (70,0%)	5 (25,0%)	1 (5,0%)	0 (0,0%)	
Fibrosis	1995	67 (61,5%)	22 (20,2%)	16 (14,7%)	4 (3,7%)	0,173
	2010	8 (40,0%)	6 (30,0%)	6 (30,0%)	0 (0,0%)	
Stoppers	1995	107 (98,2%)	2 (1,8%)	0 (0,0%)	0 (0,0%)	0,026
	2010	17 (85,0%)	3 (15,0%)	0 (0,0%)	0 (0,0%)	
Necrosis	1995	91 (83,5%)	18 (16,5%)	0 (0,0%)	0 (0,0%)	0,057
	2010	18 (90,0%)	1 (5,0%)	1 (5,0%)	0 (0,0%)	
Apoptosis	1995	109 (100,0%)	0 (0,0%)	0 (0,0%)	0 (0,0%)	< 0,001
	2010	4 (20,0%)	8 (40,0%)	7 (35,0%)	1 (5,0%)	
Increased Islets	2010 1995	8 (40,0%) 109 (100,0%)	9 (45,0%) 0 (0,0%)	3 (15,0%)0 (0,0%)	0 (0,0%) 0 (0,0%)	< 0,001
Islets with	1995	109 (100,0%)	0 (0,0%)	0 (0,0%)	0 (0,0%)	0,023
dysplasia-like changes	2010	18 (90,0%)	2 (10,0%)	0 (0,0%)	0 (0,0%)	

(*) Descriptive level of probability of Fisher’s exact test.

The vast majority of patients in the HAART group did not present dysplasia-like alterations whereas in the non-HAART group there were 48 cases with mild degree and 19 moderate degree cases. The groups differed in relation to atrophy: the HAART group had comparatively a higher frequency in the intense degree, with 2 out of 20 cases whereas the non-HAART group had only 3 out of 109 cases.

The groups differed in relation to secretion inhibitors: the HAART group had 3 out of 20 cases with mild degree whereas the non-HAART group had only 2 out of 109 cases (p<0.026). The groups also differed on apoptosis and increased islets. The non-HAART group did not present this abnormality and the HAART group showed different degrees of abnormalities (p<0.001).

The groups also showed differences in relation to islets with dysplasia-like changes. The HAART group presented a higher rate of mild cases and a lower rate of absent cases when compared to the non HAART group.

In Table 4 we observe that the groups differed in the presence of foci of linfomononuclear inflammatory infiltrate. The majority of the cases in the HAART group, 12 out of 20, presented this infiltrate. On the other hand, the non-HAART group presented only 9 out of 109 cases.

**Table 4:** Acute pancreatitis, foci of lymphocytic infiltrate and opportunistic infections detected on autopsy in the year1995 (n= 109) and 2010 (n = 20)

**Presence of features**	**1995 (n=109)**		**2010 (n=20)**		p*
	**n**	**%**	**n**	**%**	
Acute pancreatitis	10	9,2	0	0,0	0,360
Foci of lymphocytic in filtrate	9	8,3	12	60,0	<0,001
Mycobacteria	21	19,3	2	10,0	0,525
Fungi	4	3,7	1	5,0	0,576
Bacteria	10	9,2	0	0,0	0,360
Protozoa	16	14,7	0	0,0	0,131

(*) descriptive level of probability of Fisher’s exact test.

In order to investigate possible opportunistic infections, an immunohistochemistry assay for common pathogens (bacteria, fungi, protozoa and mycobacteria) was performed, but no statistical differences between the groups were found (Table 4).

## Discussion

When analyzing the last ten years of treatment for AIDS, it is remarkable how life expectancy has increased due to the introduction of the highly active antiretroviral therapy and its easy access to the general population. This is a fact that helped to reduce death rates related to AIDS in 2010, justifying the sample of only 20 cases in the HAART group. The period to obtain 20 cases to accomplish this study was from June 2006 to December 2009. The samples obtained in 1995 encompassed 109 cases, probably due to a higher number of deaths (without potent antiviral therapy) and the unawareness of the disease and its consequences.

In this period of 10 years the comparative sample of the pancreas in 129 cases (109 cases in 1995 and 20 cases in 2010) was characterized by similarities of histological findings and ethnicity, contrary to author’s expectations. There was homogeneity among samples, despite the 10-year gap between groups.

The average age of the groups is in agreement with data from the Ministry of Health and UNAIDS concerning to males [1,2]. These data were not expected once the AIDS epidemic has been turning to females. Nevertheless, the 2010 population is still predominantly composed of males and older individuals. This may be due to the fact the source of our patients has been the Death Verification Service of São Paulo, where autopsies are performed on patients who die of uncertain causes, probably without close monitoring in the health centers, and with poor adherence to antiretroviral treatment. From 1980 to June 2010, there were 385,815 (65.1%) infections of males against 207,080 (34.9%) females according to the Bulletin of the Ministry of Health in 2010 [2].

The Bulletin of the Brazilian Ministry of Health bears records of the increasing rates of the disease among women, especially housewives. However, the Ministry of Health now speaks about a feminization of the epidemic in the country, since the difference between men and women is shrinking. The number of cases in young people from 13 to 19 years of age increased, and this is the only age group in which the number of AIDS cases is higher among women [2].

The comparison of the data in relation to weight of the pancreas in Table 1 showed that the groups are different. The HAART group presents pancreas weight values lower than the ones from the group without drugs, maybe a consequence of longer survival and the possible effects of larger glandular depletion generated by the long use, malnutrition and the drug action that compromises the pancreas functioning.

In Table 2, concerning general manifestations, alcoholism, smoking, pancreatic manifestations and drugs used in AIDS treatment, fever occurred more frequently in the non-HAART group when compared to the HAART group, probably because they did not make use of antiretroviral drugs and became more susceptible to opportunistic infections.

As for the weight loss, there was no difference between the groups. Individuals with HIV develop two models of malnutrition: protein-caloric and wasting syndrome which cause weight loss. Severe malnutrition reduces survival and jeopardizes the quality of life in the HIV positive individual

Regarding the diarrhea described in Table 2, there was no significant difference between the two groups. The causes of diarrhea may be due to drug actions, changes in the intestinal luminal fluid tonicity, changes of the transport processes of electrolytes and water through the intestinal epithelium, undigested and unabsorbed nutrients because of the increase of the tonicity of the intestinal lumen in relation to the plasma, causing diarrhea of osmotic type and infectious processes by various pathogens [14-17].

In our sample, the side effects of antiretroviral drugs implied electrolyte imbalance and water absorptive process, a probable cause of diarrhea in the HAART group in relation to the non-HAART group.

The prevalence of increased abdominal pain in the HAART group may be associated with smoking and drinking, current in the HAART group compared to the group without HAART. These are factors that cause lesions such as chronic pancreatitis as well as pancreatic fibrosis.

Table 3, concerning the semi-quantitative microscopic aspect of acinar components of the pancreatic stroma and histological aspects of acini, shows that the decrease of the zymogen granules, the decrease of core dysplasia-like, the important increase of pancreatic atrophy, the increase of apoptosis and the maintenance of the parenchymal steatosis are more evident in the HAART group. These effects may represent a longer survival, keeping the gland protected from the drugs, agents and other damaging factors such as alcohol. With the reduction of the enzymes and atrophy of the parenchyma, we can assume that these patients may develop the malabsorption syndrome, represented by steatorrhea.

In the treated group, another surprising change was observed due to great abnormalities in the islets of Langerhans represented by the increase of number, volume and dysplastic nuclei. These findings must be compatible to the longer use of antivirals.

Changes in the islets of Langerhans by a possible action of the antiretroviral therapy may increase insulin secretion and glucose uptake by cells of the body as an energy source, increasing the concentration of glucose in the blood and thus causing diabetes. According to a study by Monteiro [18] it is reported that profound metabolic changes in HIV positive patients are associated with the use of antiretroviral drugs like Stavudine, Indinavir and Lopinavir with Ritonavir. This mechanism can cause a change in insulin secretion in the HIV positive individual [18].

In Table 4, concerning the acute pancreatitis, foci of lymphocytic inflammatory infiltrate and opportunistic infections were detected at necropsy, foci of lymphocytic inflammatory infiltrate predominated in irregular and multifocal parenchymal gland. These infiltrates were caused by ductal changes resulting from the pancreas necrotic inflammatory process. As a consequence, alterations in the exocrine and endocrine gland functions increase the diabetes rate, especially in the HAART group, probably due to the high rates of alcoholism.

Regarding the immunohistochemical reactions for the characterization of the infectious agents, the CMV (*Cytomegalovirus)* and the PMC (*Pneumocystis carinii*) showed no differences in both groups. It was observed that the treated group presented probable immunological reconstitution with a significant decrease of opportunistic infections.

## Conclusions

The analysis of the sample population of AIDS patients showed no differences in the two groups or the ones reported in other national and international centers, regarding age, sex and race.

In conclusion, no evidence of pancreatic lesions secondary to antiretroviral therapy was found because of the similarity of the histological findings between samples, in spite of the time interval and the use of highly active antiretroviral therapy. They are probably due to nutritional factors and/or related to HIV virus infection and its complications.

## Competing interests

The authors declare there are no conflict of interests and no source of funding.

## Authors’ contributions

EZC: conducted the study. FLAF: made the laboratorial analysis and conducted the study. AGB: collected the data and performed all the interviews. AAM: made all the pathology analysis. MRB: wrote the article and the tables. The present statement certifies that all the author’s have read the paper and approved its final version.

## References

[B1] UNAIDS Report on the global AIDS epidemic 2010 [http://www.unaids.org/globalreport]

[B2] Brazilian AIDS Bulletin 2010 [http://www.aids.gov.br/sites/default/files/anexos/publicacao/2010/45974/boletim_dst_aids_2010_pdf_19557.pdf]

[B3] Brivet F Coffin B Bedossa P Pancreatic lesions in AIDS Lancet 1987 8558 570 571 2887861 10.1016/s0140-6736(87)92956-4

[B4] Chehter EZ Mott CB Uip DE AIDS and pancreas: a retrospective study Proceedings of the 10th World Congress of Gastroenterology 1994 Los Angeles: Abstract Book

[B5] Chehter EZ Acquired Immunodeficiency Syndrome (AIDS) and pancreas: a prospective study of clinical and pathological features 1997 Gastroenterology Department: PhD tesis. Faculdade de Medicina da USP

[B6] Chehter EZ Longo MA Laudanna AA Involvement of the pancreas in AIDS: a prospective study of 109 post-mortems AIDS 2000 14 1879 1886 10.1097/00002030-200009080-00001 10997390

[B7] Balani AR Grendell JH Drug-Induced Pancreatitis Drug Safety 2008 3 10 823 837 18759507 10.2165/00002018-200831100-00002

[B8] Riedel DJ Gebo KA Moore RD A Ten-Year Analysis of the Incidence and Risk Factors for Acute Pancreatitis Requiring Hospitalization in an Urban HIV Clinical Cohort AIDS Patient Care STDS 2008 22 2 113 121 10.1089/apc.2007.0034 18260802 PMC2561218

[B9] Chehter EZ Duarte MIS Takakura CFH Ultrastructural Study of the Pancreas in AIDS Pancreas 2003 26 2 153 159 10.1097/00006676-200303000-00011 12604913

[B10] Kerr JFR Wyllie AH Currie AR Apoptosis: a basic biological phenomenon with wide-ranging implications in tissue kinetics BR J Cancer 1972 26 239 257 10.1038/bjc.1972.33 4561027 PMC2008650

[B11] Michalany J Histhological technique in pathological anatomy: with instructions to surgeon, nurse and cytotechnique 1998 Säo Paulo: Publisher Michalany 3

[B12] Bradley EL A clinically based classification system for acute pancreatitis: summary of the international symposium on acute pancreatitis. Atlanta 1992 ArchSurg 1993 128 586 590 10.1001/archsurg.1993.01420170122019 8489394

[B13] Kloppell G Maillet B Pathology of acute and chronic pancreatitis Pancreas 1993 8 659 670 10.1097/00006676-199311000-00001 8255882

[B14] Focaccia R Veronesi R Textbook of Infectious Diseases Gastrointestinal manifestations 2009 São Paulo: Atheneu 208 224

[B15] Guyton AC Textbook of Medical Physiology Physiology of the Gastrointestinal disorders 2006 Rio de Janeiro: Elsevier 822 823

[B16] Douglas CR Textbook of Physiology applied to the medical sciences Physiology of the small intestine 2006 Rio de Janeiro: Guanabara Koogan 934

[B17] Robbins C Pathologic Basis of Disease The Gastrointestinal Tract 2005 Rio de Janeiro: Elsevier 873 874

[B18] Caseiro MM Metabolic Abnormalities[http://www.saberviver.org.br/index.php?g_edicao=alteracoes_metabolicas]

